# A mitochondria‐targeted aggregation‐induced emission photosensitizer for eradication *Candida* bioﬁlms and treating oral ulcer

**DOI:** 10.1002/smo.20240060

**Published:** 2025-02-17

**Authors:** Kun‐Mei Liu, Yun Wang, Feng‐Wei Xia, Shun Feng, Xiao‐Qi Yu, Ming‐Yu Wu

**Affiliations:** ^1^ College of Biomedical Engineering Sichuan University Chengdu China; ^2^ School of Life Science and Engineering Southwest Jiaotong University Chengdu China; ^3^ Asymmetric Synthesis and Chiral Technology Key Laboratory of Sichuan Province Department of Chemistry Xihua University Chengdu China

**Keywords:** aggregation‐induced emission, biofilm, *candida* infection, mitochondrial targeting photosensitizer, photodynamics therapy

## Abstract

With increasing drug resistance, *Candida* infections have posed serious threats to public health. Photodynamic therapy harnesses light to destroy pathomycete, providing a smart strategy for combating of *Candida* infections. However, due to lack of organelle targeting ability and bad extracellular polymeric substances penetrability, current photosensitizers (PSs) are far from desirable to clean biofilms and fight against drug resistance. Herein, a mitochondrion targeting aggregation‐induced emission PS, LIQ‐TPA‐TZ, was developed for the efficient photodynamic treatment of oral *Candida* infection. LIQ‐TPA‐TZ has good singlet oxygen and hydroxyl radical generation ability, which can efficiently kill the *Candida guilliermondii* (*C. guilliermondii*) and eradicate the biofilm. It not only causes mitochondrial damage by disruption of mitochondrial respiratory chain and oxidative stress‐related gene but also inhibits fungal adhesion and filamentous growth to prevent *Candida* colonization, mycelia growth and biofilm formation, which is favorable for eliminating the potential drug resistance. In the mouse oropharyngeal *Candida* biofilm infection model, LIQ‐TPA‐TZ significantly eliminates infection, alleviates inflammation, and accelerates mucosal defect healing. This study provides a favorable strategy for confronting drug resistance, which may be a potential Candidate for the treatment of *Candida* infection.

## Graphical abstract

A mitochondria‐targeted AIE photosensitizer, LIQ‐TPA‐TZ, was developed to fight against *Candida* infection. It can efficiently produce reactive oxygen species to kill *Candida guilliermondii* and eradicate biofilms. It not only disrupted the respiratory chain and affected the metabolism and energy supply but also reduced the fungi adhesion and filament growth capabilities to conquer biofilm resistance.

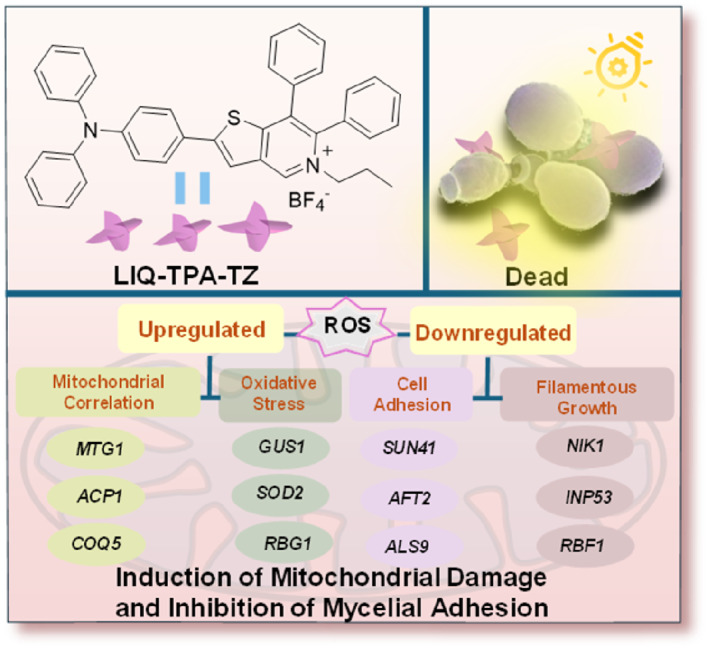

## 1 INTRODUCTION

Oral candidiasis is a common fungal infection with a global prevalence of about 5%–7%, especially in immunosuppressed patients, pregnant women, and people who use drugs chronically.[^1^, ^2^, ^3^] *Candidas* are chief culprits, which take full advantage of the enzymes secreted by them to destroy the epithelium barrier of the host cells, change to the mycelium morphology and invade deep tissues, causing inflammatory response.[^4^, ^5^, ^6^, ^7^] Consequently, they can lead to painful ulcers in the oral mucosa that affect eating and speech, which further cause dysphagia and even spread to the esophagus and the whole body, leading to more serious systemic infections. Clinically, antifungal drugs such as clotrimazole and fluconazole are the first choice. However, long‐term abuse of antibiotics may lead to increasing fungal resistance as well as obvious side effects.[^8^, ^9^, ^10^] Furthermore, *Candida* tends to change its morphology to form pyknotic biofilm to resist to antibiotics and immune defenses, resulting in 10–1000 folds higher drug resistance.[^11^, ^12^] In view of the growing fungal infection, the development of new antifungal method is particularly urgent.

Photodynamic therapy (PDT) harnesses light to generate reactive oxygen species (ROS) by leveraging photosensitizers (PSs) to destroy microbial cell structures, such as lipids, nucleic acids and proteins, providing new and smart ideas for combating of candidiasis infections with the advantages of minimal drug resistance, diminished systemic toxicity and low side effects.[^13^, ^14^, ^15^, ^16^, ^17^, ^18^] Therefore, a lot of PSs have been developed for fighting against fungal infections.[^19^, ^20^, ^21^] In particular, PSs with aggregation‐induced emission (AIE) properties, which can reduce nonradiative decay and the energy gap of the intersystem crossing in the aggregate state as compared to that in a discrete molecular state, resulting in increased ROS production, have attracted more and more attention.[^22^, ^23^, ^24^, ^25^] However, the antifungal efficacy and antibioflim capacity of these PSs are far from ideal due to the lack of targeting capabilities and bad extracellular polymeric substances penetrability.

The therapeutic efficacy of PS is greatly influenced by the short diffusion distance and lifespan of ROS.[^26^, ^27^] The targetability of PS plays a crucial role in determining its effectiveness. Organelles are vital for maintaining fungal functions and are involved in a range of cellular processes. Targeting PSs to specific organelles can significantly enhance PDT efficiency.[^28^, ^29^] Among the organelles, mitochondria not only play a central role in aerobic respiration and energy supply but also are crucial in fungal morphogenesis, virulence, and drug‐resistance.[^30^, ^31^, ^32^] Furthermore, the powerful antioxidant defense system in the fungal cell is closely related to mitochondria.^[33]^ The superal oxide dismissal enzyme, glutathione peroxidase and sulfur oxygen protein system (Trx/TrxR) in the mitochondria can effectively remove ROS to generate new drug resistance to PDT.[^34^, ^35^, ^36^, ^37^] Synthetically, these make mitochondria an attractive target for fungicide development. Two of the market leader single‐target site fungicides, the SDHIs and strobilurins, disrupt the fungal mitochondrial respiration chain.[^38^, ^39^, ^40^]

Due to its unique structural and biological activity, isoquinoliniums have extensive application prospects in antitumor, antiviral and antibacteria.^[41]^ In our previous studies, we systematically constructed isoquinolinium based AIE PSs for high fidelity imaging and long‐term tracking mitochondria through a convenient Rh‐catalyzed tandem reaction strategy.[^42^, ^43^, ^44^] For improving the ROS generation efficiency, we modified the conjugated system from phenyl to furan to thiophene, meanwhile, the different electron donor was adopted to developed mitochondria targeting AIE PS for photodynamic anticancer and antibacterial therapy.

Through regulation the hydrophilic and hydrophobic balance of cationic isoquinolinium, LIQ‐TPA‐TZ was obtained, which can overcome the physical barrier of fungal cell wall and membrane, specifically targeted the mitochondria of *Candida* and destroyed the oxidative defense system of mitochondria by activating ROS production through light, leading to mitochondrial damage and inducing *Candida* death to minimize the development of potential drug resistance. LIQ‐TPA‐TZ‐mediated PDT could promote the expression of mitochondrial respiratory chain and oxidative stress‐related gene in *Candida*, causing damage to the inner and outer membrane components, and interfering membrane potential homeostasis. Moreover, it also reduced the expression of genes related to adhesion and filamentous growth to inhibit the colonization of *Candida*, thereby affecting the growth of mycelia and the ability to form biofilms. In vivo, it destroyed the colonized mycelia, cleared the biofilm on the mucosal surface, and accelerated the healing of mucosal defects to treat oral ulcers.

## 2 RESULTS AND DISCUSSION

### 2.1 Photophysical properties of LIQ‐TPA‐TZ

**FIGURE 1:**
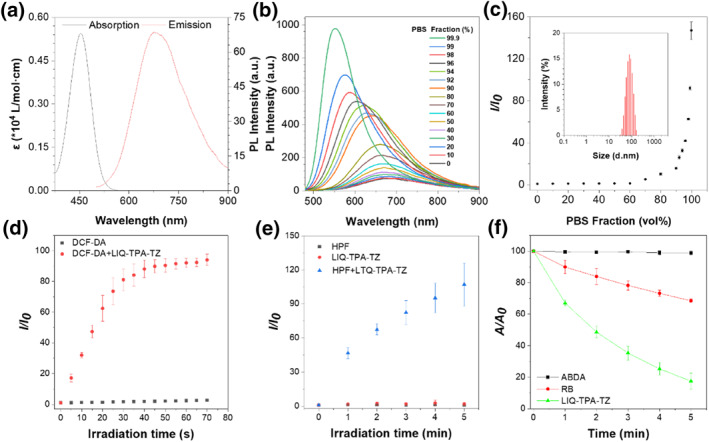
Photophysical properties of LIQ‐TPA‐TZ. (a) Molar absorption coefficient and emission spectra of LIQ‐TPA‐TZ in DMSO. (b) PL spectra of LIQ‐TPA‐TZ (10 μM) in mixtures of DMSO and PBS with different PBS contents. (c) Plot of the relative emission intensity of LIQ‐TPA‐TZ in DMSO with different PBS fractions, *I*_0_ and *I* are the peak values of PL intensities of LIQ‐TPA‐TZ (10 μM) in DMSO and DMSO/PBS mixtures, respectively. Inset: size distribution of LIQ‐TPA‐TZ in aggregated (99.9% PBS). (d) ROS generation of LIQ‐TPA‐TZ upon white light (20 mW cm^−2^) irradiation in PBS solution. (e) •OH generation of LIQ‐TPA‐TZ upon white light (20 mW cm^−2^) irradiation in PBS solution. (f) Decomposition rates of ABDA in the absence or presence of LIQ‐TPA‐TZ or RB under white light (20 mW cm^−2^) illumination in water. All experiments in d, e and f were repeated three times. ABDA, anthracenediyl‐bis(methylene) dimalonic acid; DMSO, dimethyl sulfoxide; •OH, hydroxyl radical; PBS, phosphate buffered saline; PL, photoluminescence; RB, rose bengal; ROS, reactive oxygen species.

The synthesis of LIQ‐TPA‐TZ refers to our previous work.^[44]^ The photophysical properties were investigated with ultraviolet and visible spectrophotometer and photoluminescence spectrophotometer (Figure 1 and Table S1 in Supporting Information S1). LIQ‐TPA‐TZ showed a maximum absorption peak at 454 nm in dimethyl sulfoxide (DMSO) solution with a molar absorption coefficient of 0.544 × 10^4^ L mol^−1^ cm^−1^. The maximum emission peak was observed at 680 nm with a Stokes shift of 226 nm (Figure 1a). Subsequently, the DMSO/phosphate buffered saline (PBS) system was chosen to evaluate the AIE properties. When the PBS fraction was below 80%, LIQ‐TPA‐TZ displayed minimal luminescence with a quantum yield (QY) of 2.23% (Figures 1b–c). However, increasing the PBS fraction beyond 90% led to the formation of aggregates, which significantly intensified the emission. Amazingly, in 99.9% PBS, the fluorescence intensity was 146.9‐fold higher than that in pure DMSO solution, and the QY increased to 9.38%. Dynamic light scattering analysis confirmed the presence of aggregates with an average diameter of approximately 230 nm in the 99.9% PBS solution (Figure 1c).

The ROS‐generating capability of LIQ‐TPA‐TZ was evaluated using 2′,7′‐dichlorodihydrofluorescein (DCFH) as an indicator. As depicted in Figure 1d and S1 in Supporting Information S1, the fluorescence intensity of DCFH increased by more than 100‐fold within 70 s of continuous irradiation in the presence of LIQ‐TPA‐TZ. In contrast, negligible fluorescence enhancement was observed after irradiating the solution without LIQ‐TPA‐TZ, demonstrating the high ROS generation efficiency of LIQ‐TPA‐TZ.

Subsequently, hydroxyphenyl fluorescein was utilized to assess the efficiency of hydroxyl radical (•OH) generation. As illustrated in Figure 1e and S2 in Supporting Information S1, in the absence of LIQ‐TPA‐TZ, hardly any fluorescence increase was detected under white light irradiation. In contrast, it increased by more than 100‐fold after 5 min of continuous irradiation in the presence of LIQ‐TPA‐TZ, indicating that it can effectively produce •OH via the Type I mechanism.

Additionally, 9,10‐anthracenediyl‐bis(methylene) dimalonic acid (anthracenediyl‐bis(methylene) dimalonic acid (ABDA)) was employed to measure the generation of singlet oxygen (^1^O_2_). Under light irradiation, the absorbance of ABDA hardly changed. However, after adding commercially available PS and rose bengal (RB), more than 48% ABDA was decomposed. In comparison, the absorption of ABDA decreased by approximately 84% after treatment with LIQ‐TPA‐TZ, demonstrating the outstanding ^1^O_2_ generation performance (Figure 1f and S3 in Supporting Information S1). The above results demonstrated that LIQ‐TPA‐TZ can highly efficiently generate •OH and ^1^O_2_ through both type I and type II mechanisms. Biofilms are heterogeneous, with oxygen‐enriched surfaces and hypoxic in the interior. The ability to generate a different types of ROS on‐demand could efficiently address the limitations of single‐type PS in different temporal and spatial conditions.

### 2.2 In Vitro antifungal effect of LIQ‐TPA‐TZ

To evaluate the potential of LIQ‐TPA‐TZ as a photodynamic antifungal agent, we first assessed its biocompatibility through hemolytic tests. As shown in Figure S4A and B in Supporting Information S1, the positive control group showed ruptured blood cells and released hemoglobin, whereas PBS did not induce any hemolysis of red blood cells. Even at the concentration of 16 μM, no significant hemolysis was observed for LIQ‐TPA‐TZ. The Cell Counting Kit‐8 assay was used to assess the biocompatibility. As illustrated in Figure S4C in Supporting Information S1, over 90% of mouse fibroblast (L‐929) cells still survived after treatment with 16 μM of LIQ‐TPA‐TZ, whenever in the dark or exposed to light irradiation. These results highlighted the excellent biocompatibility of LIQ‐TPA‐TZ.

*Candida guilliermondii* (*C. guilliermondii*) is highly pathogenic in clinical and can cause many types of infections, such as onychomycosis, acute osteomyelitis, suppurative arthritis, endocarditis, and fungemia. We examined the photodynamic antifungal efficacy of LIQ‐TPA‐TZ using a plate counting method using *C. guilliermondii* as the model.

**FIGURE 2:**
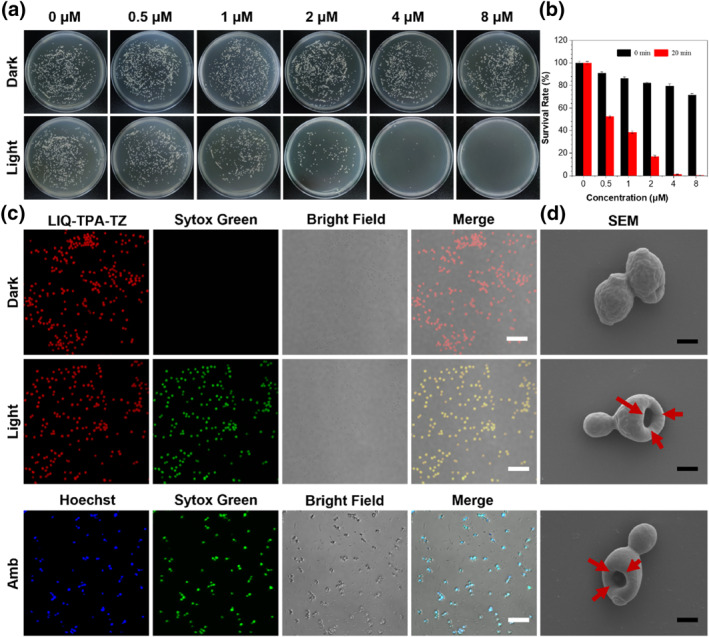
In vitro antifungal effect of LIQ‐TPA‐TZ. (a) Plates images of *C. guilliermondii* after treatment with LIQ‐TPA‐TZ for 5 min without or with light (20 mW cm^−2^) illumination for 20 min. (b) Antifungal activity of LIQ‐TPA‐TZ toward *C. guilliermondii*. (c) Fluorescence imaging of *C. guilliermondii* after incubation with 10 μM LIQ‐TPA‐TZ or Amb for 5 min, treatment without or with white light (20 mW cm^−2^) for 20 min and then stained with Sytox Green. White line indicates the scale bar (10 μm). (d) SEM images of *C. guilliermondii* after treatment with LIQ‐TPA‐TZ or Amb in the absence or presence of white light (20 mW cm^−2^) illumination for morphological analysis. Black line indicates the scale bar (1 μm).

As shown in Figures 2a,b, the fungus grew smoothly on the agar plate in the absence of LIQ‐TPA‐TZ or white light irradiation. However, under white light irradiation, the number of colonies on the plate decreased significantly with the increase in the concentration of LIQ‐TPA‐TZ. When the concentration increased from 0.5 to 4 μM, the survival rate of *C. guilliermondii* decreased from 56% to 0.5%. 8 μM LIO‐TPA‐TZ could almost kill all *C. guilliermondii*.

Fluorescence imaging was conducted. Sytox Green was used to label the dead fungi (Figure 2c). Amphotericin B (Amb) was used as a positive control. After 5 min incubation with LIQ‐TPA‐TZ, all *C. guilliermondii* emitted red fluorescence. Upon light treatment, *C*. *guilliermondii* showed strong green fluorescence from Sytox Green. Meanwhile, the fungus treated with Amb can also be lit up by Sytox Green, indicating that LIQ‐TPA‐TZ effectively inactivated *C*. *guilliermondii* under light irradiation.

Scanning electron microscopy (SEM) was employed to investigate the morphology changes of *C. guilliermondii* with different treatments. As shown in Figure 2d and S5 in Supporting Information S1, the fungus showed oval‐rounded shapes with dense and homogeneous cytoplasm, intact and well‐defined cell walls, and clear membrane boundaries. However, after treatment with LIQ‐TPA‐TZ and light irradiation or Amb, the fungal cells were concave with holes. These results conclusively demonstrated the exceptional performance of LIQ‐TPA‐TZ in photodynamic killing *C. guilliermondii*.

### 2.3 In Vitro antibiofilm effect of LIQ‐TPA‐TZ

**FIGURE 3:**
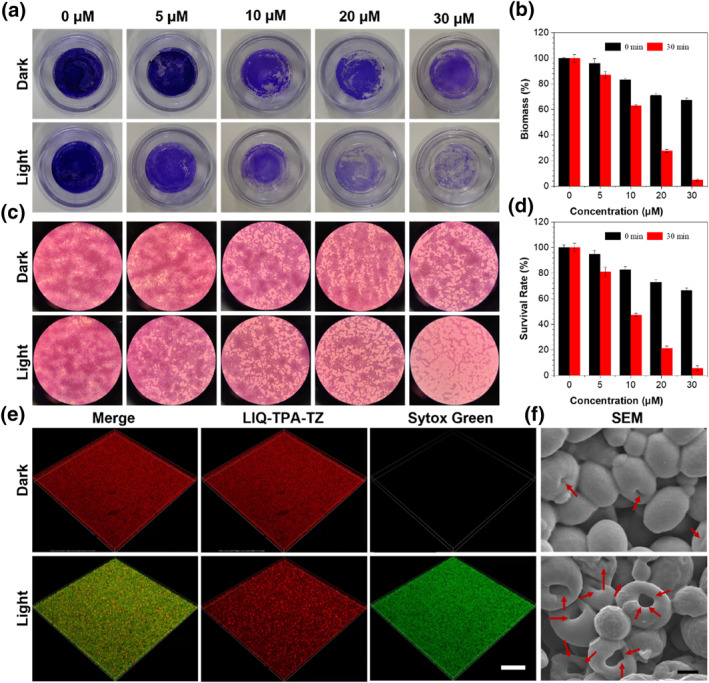
In vitro antibiofilm effect of LIQ‐TPA‐TZ. Crystal violet‐stained images (a) and microscopic images (c) of *C. guilliermondii* biofilms formed on culture dishes after treatment with different concentrations of LIQ‐TPA‐TZ under dark or white light white light (20 mW cm^−2^) irradiation. (b) Biofilm biomass of *C. guilliermondii* treated with different concentration of LIQ‐TPA‐TZ under dark or irradiated with light (20 mW cm^−2^). (d) Survival rate of *C. guilliermondii* in the biofilm treated with different concentration of LIQ‐TPA‐TZ under dark or irradiated with light (20 mW cm^−2^). (e) Representative 3D fluorescence images of *C. guilliermondii* biofilms after incubated with 30 μM LIQ‐TPA‐TZ for 30 min, treated without or with white light (20 mW cm^−2^) for 30 min and then stained with Sytox Green. White line indicates the scale bar (50 μm). (f) Representative SEM images of *C. guilliermondii* biofilms with different treatments. Biofilms were cocultured with 30 μM LIQ‐TPA‐TZ and then kept in the dark or irradiated with white light (20 mW cm^−2^) for 30 min. Black line indicates the scale bar (1 μm).

*Candida*s tend to form biofilms to resist both antifungal agents and the host's immune response to increase the drug resistance as high as 10–1000 folds. The persistence of biofilms as a source of infection underscores the need for effective antibiofilm strategies. To assess the effectiveness of LIQ‐TPA‐TZ mediated PDT to eliminate biofilm, crystal violet staining was carried out. As shown in Figure 3a, *Candida* biofilm was intact whenever under dark or light conditions. Nevertheless, under light irradiation, when the concentration of LIQ‐TPA‐TZ increased from 0 to 30 μM, *Candida* biofilm was broken and ruptured obviously. Besides, with the increased concentration of LIQ‐TPA‐TZ, the blue color of crystal violet in the light group became thinner than that in the dark group, indicating that the biofilm decreased. When treated with 30 μM LIQ‐TPA‐TZ, there was almost no *Candida* biofilm (Figure 3c).

The thickness of the biofilm was largely determined by the ability of fungi to adhere to the biofilm, and the reduction of the biofilm thickness indicated that the bacteria were shed from the biofilm. As shown in Figure 3b, when the LIQ‐TPA‐TZ concentration increased from 5 to 30 μM, the biomass of the biofilm decreased from 84% to 3%. The adhesion ability of *Candida* to biofilm was significantly decreased, thus inhibiting the formation of biofilm. Furthermore, the methylthiazolyldiphenyl‐tetrazolium bromide assay was used to determine the survival rate of *C. guilliermondii* in the biofilm. As shown in Figure 3d, with the help of light, the surviving *Candida* in biofilm gradually decreased from 83% to 5%, respectively, when the concentration of LIQ‐TPA‐TZ increased from 5 to 30 μM.

Furthermore, the antifungal efficacy of LIQ‐TPA‐TZ in biofilms was evaluated using confocal laser scanning microscopy. As illustrated in Figure 3e, a pronounced green fluorescence from Sytox Green was observed in the biofilm treated with LIQ‐TPA‐TZ and light irradiation, indicating that the fungus in the biofilm was dead. In contrast, hardly any green fluorescence was observed in the biofilm when kept in the dark. Additionally, SEM was utilized to assess the morphological changes of fungi within biofilms. As depicted in Figure 3f and S6 in Supporting Information S1, the control group showed healthy fungi with a uniformly smooth surface, whenever in the presence or absence of light. However, after treatment with Amb or LIQ‐TPA‐TZ and light irradiation, all the fungi displayed deformations, depressions, and noticeable surface pores. These results fully demonstrated that LIQ‐TPA‐TZ‐mediated PDT can destroy the biofilm and kill the fungus inside the biofilm.

### 2.4 Potential antifungal mechanism of LIQ‐TPA‐TZ

**FIGURE 4:**
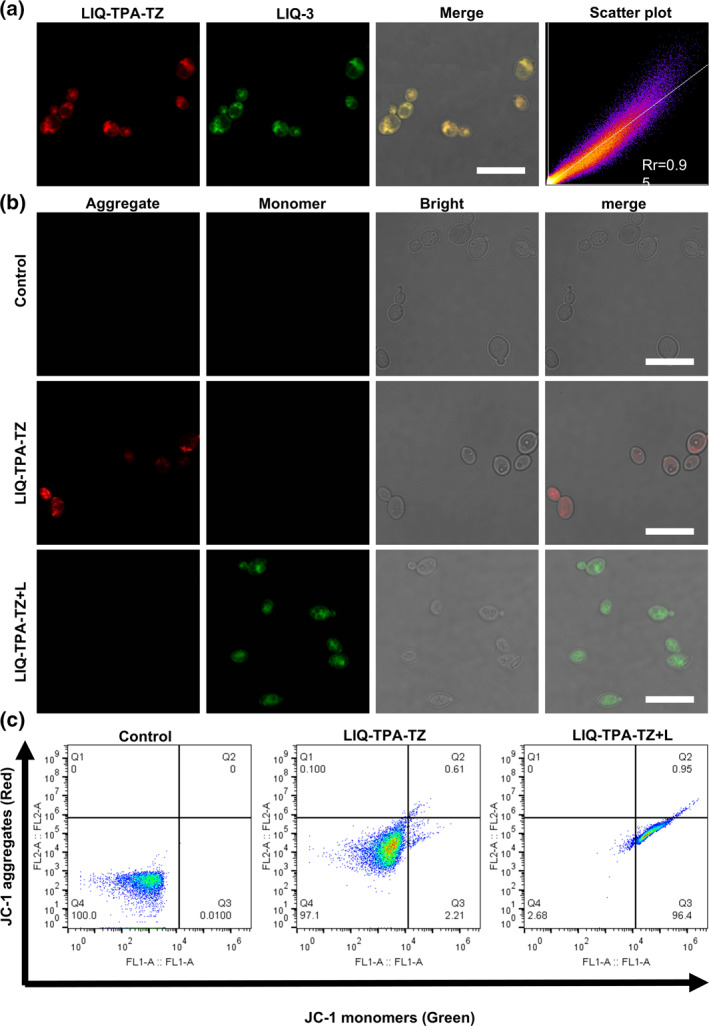
Potential antifungal mechanism of LIQ‐TPA‐TZ. (a) Co‐localization of LIQ‐TPA‐TZ with mitochondrial targeting probe LIQ‐3 in *C*. *guilliermondii*. Green fluorescence was from LIQ‐3 (*λ*_ex_ = 405 nm, *λ*_em_ = 500–600 nm); Red fluorescence was from LIQ‐TPA‐TZ (*λ*_ex_ = 450 nm, *λ*_em_ = 600–720 nm). (b) Fluorescence images of MMP changes with JC‐1. Scale bar: 10 μm. (c) Flow cytometry analysis of MMP after treatment with LIQ‐TPA‐TZ under dark or light (20 mW cm^−2^) irradiation. MMP, mitochondrial membrane potential.

The mitochondrial targeting ability of LIQ‐TPA‐TZ was first investigated. After incubation with LIQ‐TPA‐TZ, *C*. *guilliermondii* was counterstained with the mitochondria targeting fluorescent probe. As depicted in Figure 4a, the fluorescence signals of LIQ‐TPA‐TZ overlapped well with LIQ‐3. The Pearson correlation coefficient (Rr) values were as high as 0.95, indicating that LIQ‐TPA‐TZ can selectively accumulate in the mitochondria.

Given that LIQ‐TPA‐TZ is predominantly localized in fungal mitochondria, it was postulated that the production of ROS within mitochondria would impair their integrity, ultimately leading to fungal death. It is known that mitochondrial membrane potential (MMP) collapse signifies mitochondrial dysfunction and damage. Therefore, the MMP was monitored using the MMP indicator, JC‐1, through fluorescence imaging and flow cytometry. Under dark conditions, the red fluorescence of the fungi was intensified following the co‐culture of fungi with LIQ‐TPA‐TZ and JC‐1. However, after further treatment with white light irradiation for 20 min, the red fluorescence disappeared accompanied by green fluorescence turned on, indicating that the generation of ROS by LIQ‐TPA‐TZ induced MMP destruction and mitochondrial damage (Figures 4b,c).

In addition, we conducted RNA sequencing and gene expression analysis to further investigate the antifungal mechanism. As shown in Figure S7A in Supporting Information S1, a Principal Component Analysis plot was used to assess the biological repeatability within groups and the differences between different groups. The samples of the LIQ‐TPA‐TZ group and the control group were closely clustered with each other, while the distance between the two groups was significant, indicating significant differences between the two groups and good similarity within‐group, meeting statistical requirements. Figure S7B in Supporting Information S1 showed the statistical chart of differentially expressed genes (DEGs), identifying a total of 1412 DEGs, including 662 upregulated genes and 750 downregulated genes.

**FIGURE 5:**
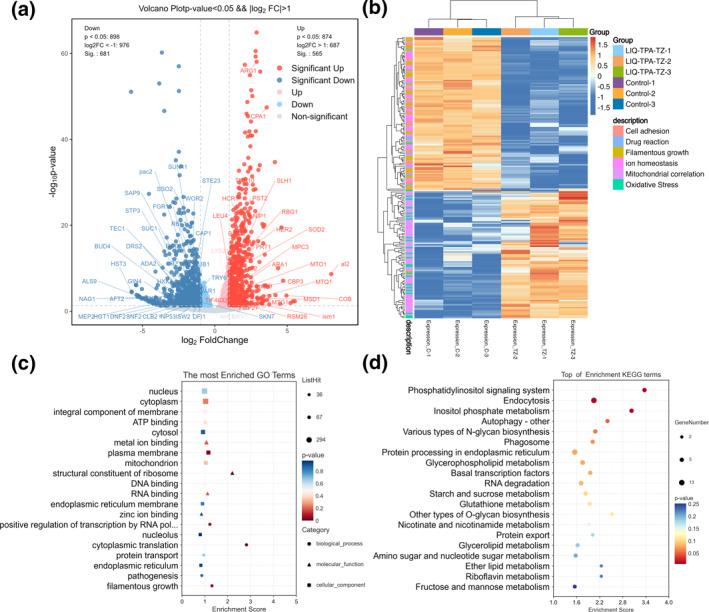
Summary of candidiasis gene transcriptome sequencing results. (a) Volcano map of significantly up‐regulated (red) and down‐regulated (blue) genes. (b) Heat map of specific gene expression, in units of log2 (FPKM). (c) GO enrichment bubble plot of differentially genes. (d) KEGG pathway enrichment analysis of differentially genes. FPKM, fragments per kilobase million; GO, gene ontology; KEGG, kyoto encyclopedia of genes and genomes.

The DEGs were categorized into 6 groups: oxidative stress, cell adhesion, filamentous growth, drug response, ion homeostasis, and mitochondrial function. The specific gene symbols for each category can be found in Table S2 in Supporting Information S1. As shown in the volcano map (Figure 5a), genes associated with oxidative stress (such as *SOD2*, *RBG1*, *GUS1*) and genes related to mitochondrial respiratory chain (such as *MTG1*, *ACP1*, *COQ5*, *COX8*) were upregulated. Conversely, genes related to cell adhesion (*SUN41*, *AFT2*, *ALS9*), filamentous growth (*NIK1*, *INP53*, *RBF1*), drug response (*CAP1*, *SKY1*, *CRZ1*), and ion homeostasis (*APE1*, *SMF1*, *ATG15*) showed downregulation. Cell adhesion and filamentous growth are closely related to the formation of the fungal biofilms. Cell adhesion downregulation indicated the initiation of biofilm formation, while filamentous growth was the major survival form of filamentous yeast cells in response to biofilm hypoxia and nutrient‐deprived environments. Drug response represents the fungal defense response to drugs.

LIQ‐TPA‐TZ treatment resulted in increased expression of genes related to oxidative stress and mitochondrial function, as shown in Figure 5b. This indicated that fungal cell stress granules were activated by ROS produced by the LIQ‐TPA‐TZ, leading to enhanced secretion of redox enzymes to counteract oxidative damage to cellular biomolecules. The increased expression of genes related to mitochondrial respiratory chain, membrane potential, composition, DNA replication, and translation indicated that the LIQ‐TPA‐TZ primarily affected fungal mitochondria, not only disrupting the respiratory chain and glycolysis but also damaging the inner and outer membrane composition, interfering with MMP homeostasis, and proliferative functions. The reduced expression of genes related to cell adhesion and filamentous growth indicated a significant impact on the ability of fungal biofilm formation. During biofilm formation, fungi need to secrete adhesion factors and spread hyphae interweaving extracellular matrix to adhere to material surfaces, which was significantly inhibited by the LIQ‐TPA‐TZ. Additionally, the weakened defense response of fungi to drugs suggested that LIQ‐TPA‐TZ damaged the fungal virulence defense state and had the potential to overcome drug resistance.

To further understand the impact of LIQ‐TPA‐TZ on biological functions, Gene ontology analysis of DEGs was carried out, classifying them into three basic functional categories: biological processes (BP), cellular components, and molecular functions (Figure 5c). In BP, genes were found to be mainly concentrated in labels such as “filamentous” and “cytoplasmic translation,” whereas MF‐related genes were enriched in functional annotations such as “metal ion binding” and “structural constituent of ribosome.” Additionally, CC‐related genes showed significant enrichment in the “plasma membrane” component, followed by the “mitochondrion.” This suggested that the PS predominantly targeted fungal plasma membranes and mitochondria, affecting the normal functioning of membrane organelles, disrupting protein synthesis and metal ion binding, ultimately disrupting cellular ion homeostasis and respiratory chain transfer.

In addition to the functional annotations of the DEGs, pathway analysis plays a significant role in genetic research. Kyoto Encyclopedia of Genes and Genomes bubble plot (Figure 5d) associated with “Endocytosis,” “Phosphatidylinositol signaling system,” “Inositol phosphate metabolism,” and “Autophagy,” further confirming the disruption of fungal substance transport function due to PS‐induced damage to cell membrane structure. Furthermore, the inhibition of phosphatidylinositol signaling and inositol phosphate metabolism was found to affect fungal signal transduction and energy metabolism. Enrichment of the fungal autophagy mechanism suggested that ROS stimulated fungi externally, disrupting normal cellular activities and homeostasis, leading to stress responses through autophagy to provide nutrients, and even activating apoptotic pathways.

A protein‐protein interaction network was established to comprehensively study the roles of these differentially altered genes in various biological processes (Figure S8 in Supporting Information S1). The entire network was divided into different clusters using the Markov Cluster Algorithm method. It was observed that DEGs were primarily concentrated in oxidative stress, mitochondrial‐related functions, DNA expression, ion homeostasis, protein synthesis, and lysosome autophagy‐related processes, indicating that LIQ‐TPA‐TZ not only affects the plasma membrane but also disrupts ion homeostasis maintained by the plasma membrane and mitochondria, severely affecting substance transport and electron transfer in the respiratory chain. Perturbation of vacuolar ion homeostasis led to an imbalance in fungal cell osmotic pressure. These mechanisms ultimately lead to impaired normal fungal function and eventual abnormal cell death.

Based on the above results, LIQ‐TPA‐TZ effectively combats resilient fungal biofilms through three different pathways: (1) During the process of PDT, LIQ‐TPA‐TZ produces toxic free radicals that damage the fungal plasma membrane, DNA, and proteins. This disruption of the internal redox balance affects processes such as substance transport and proliferation. (2) The positive charge of LIQ‐TPA‐TZ enhances its affinity for mitochondria, altering membrane potential homeostasis and osmotic balance. This interruption leads to the disruption of the fungal respiratory chain, affecting its metabolism and energy supply. (3) LIQ‐TPA‐TZ significantly reduces the fungal cell's adhesion and filamentous growth capabilities, effectively inhibiting the formation of fungal biofilms.

### 2.5 In Vivo photodynamic antimicrobial therapy of oral candidiasis

Beneficent from the excellent mitochondria targeting ability to destroy the oxidative defense system and anti‐adhesion performance to prevent mycelium growth and biofilm formation, we further investigated the in vivo potential of LIQ‐TPA‐TZ mediated PDT for oral mucosal treatment using improved castle road mice. In the experiment, infected mice were randomly divided into 3 groups:(1) negative control group treated with PBS, (2) LIQ‐TPA‐TZ + L group treated with LIQ‐TPA‐TZ‐mediated PDT, and (3) positive control group treated with antibiotic Amb. Besides, healthy mice without infection were used as comparison (Control group). After immunosuppression with prednisolone, a glucocorticoid, the oral mucosa of mice was initially infected with *C. guilliermondii*. After establishing the oral ulcer model successfully, the mice were anesthetized with isoflurane and the tongue was pulled out with hemostatic forceps. 50 μL PBS, LIQ‐TPA‐TZ (30 μM) and Amb (30 μM) were dripped onto the wound, respectively, and incubated in the dark for 30 min. The tongue ulcer surface of mice in the LIQ‐TPA‐TZ + L group was irradiated with white light (20 mW cm^−2^) for 30 min.

**FIGURE 6:**
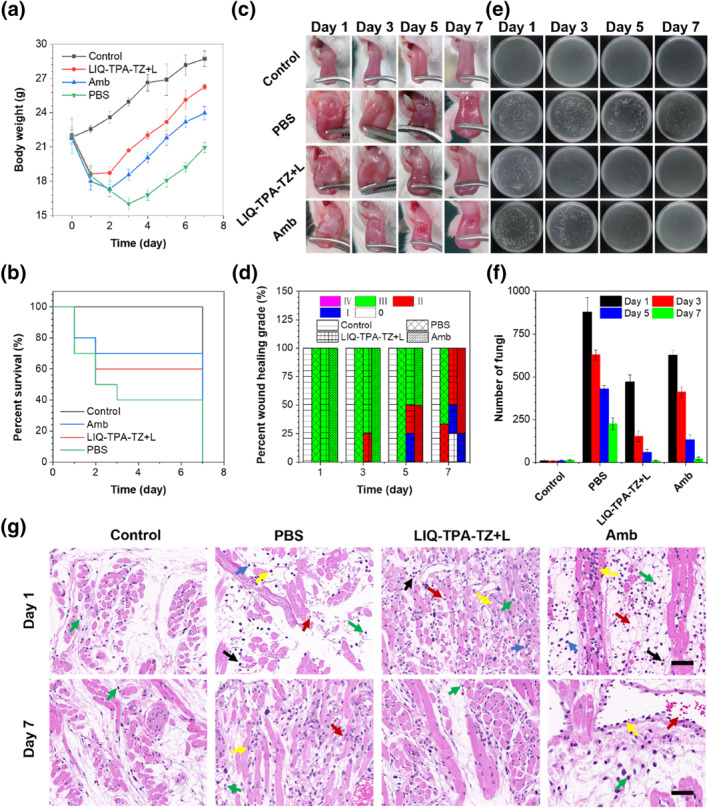
In vivo photodynamic antifungal therapy for oral candidiasis with LIQ‐TPA‐TZ. (a) Body weight changes of mice in different groups. (b) Survival rates of mice in different groups. (c) Wound healing images of mice on Day 1, 3, 5 and 7. (d) Wound healing grades of mice on Day 1, 3, 5 and 7 (data were from the corresponding images in c). (e) Images of bacterial colony obtained from tissues of mice with different treatment after 24 h of incubation. (f) Number of bacterial colonies obtained from tissues of mice treated with different treatments after 24 h of incubation (data were from the corresponding images in e). (g) H&E staining images of the tissues in different groups on day 1 and day 7. Green arrows: lymphocytes; red arrows: extravasation of red blood cells; black arrows: neutrophils; Blue arrows: cellular necrosis; yellow arrows: swelling of endothelial cells (Scale bar: 50 μm).

As shown in Figure 6a, mice without infection were healthy with increasing weight. However, due to oral mucosal infection, the mice had difficulty consuming and loss of appetite. The mice in the PBS group continued to lose weight for the first 3 days and did not return to their initial weight even after 7 days. The weight of mice in the Amb group decreased slightly after the 2 days of treatment, and gradually gained their weight at the third day. While the weight of mice in the LIQ‐TPA‐TZ + L group only decreased at the first day, and then gradually increased. 60% of mice in the PBS group died by day 3, while more than 60% and 70% mice survived in the LIQ‐TPA‐TZ + L and Amb groups (Figure 6b).

**Scheme 1:**
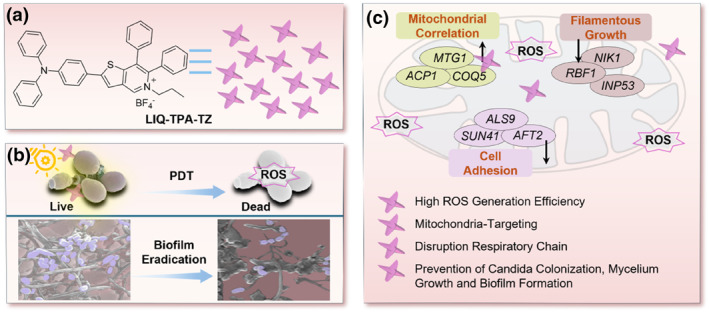
(a) Structure of **LIQ‐TPA‐TZ**. (b) Schematic diagram of photodynamic anti‐*Candida* activity and biofilm eradication of **LIQ‐TPA‐TZ**. (c) Schematic diagram of antibacterial mechanism: **LIQ‐TPA‐TZ** causes mitochondrial damage by up‐regulating the expression of genes related to mitochondrial respiratory chain, down‐regulating the expression of genes related to fungal adhesion and filamentous growth, and preventing *Candida* colonization, mycelial growth and biofilm formation.

The area of tongue ulcer, the presence of false membrane, the degree of redness and depression were recorded, which were divided into four grades. Among them, white spots, redness, ulceration, edema, etc. Occupy less than 20% of the tongue surface area is grade I; 20–50% is classed to II; 50%–90% is III; more than 90% is IV; Besides, grade 0 is normal without any of the above symptoms. The ulcer and repair of the mucous membrane on the tongue of mice with different treatments are shown in Figure 6c,d, and S9 in Supporting Information S1. The tongue surface of mice in the Control group was smooth and complete with healthy and light red, and the ulcer grade was 0. On the first day of infection, the tongues in the PBS group, LIQ‐TPA‐TZ + L group and Amb group were ulcerated and swollen, and the surfaces were covered by white false membranes. The damaged area accounted for 50%–90% of the tongue surface, and the ulcer diagnostic scores were as high as grade III. On day 3, the tongue ulcer in the PBS group was still obviously observed, and the diagnostic score was still grade III. Due to the autoimmune repair of the mice, the symptoms began to relieve on day 5, and the ulcer diagnostic score did not drop to grade II until day 7. The infection status of mice in the LIQ‐TPA‐TZ + L group improved on the day 3, and the ulcer area of the tongue was continuously reduced, and the ulcer diagnosis grade was continuously decreased. On the 7th day, 25% of the mice's tongue surface recovered with the score dropping to grade 0. In the Amb group, the wound surface began to recover on day 3, and the ulcer area gradually decreased to 50% with grade II on day 5. On day 7, the ulcer area of 25% mice dropped to grade I.

The therapeutic effects were further evaluated by counting the colonies in homogenizing wound tissue samples. As shown in Figures 6e,f, during the whole experiment, a small amount of seasonal moniliases was observed in the Control group and kept stably. On day 1, a large number of *C. guilliermondii* were present in all fungus‐infected mice, indicating that an oral ulcer mouse model was successfully constructed. Compared with the PBS and Amb groups, much fewer colonies were detected in the LIQ‐TPA‐TZ + L group on days 3 and 5. On the 7th day, the number of *Candida* colonies was similar to that in the control group, indicating that LIQ‐TPA‐TZ + L significantly inhibited the growth of fungal colonies, which was even better than Amb.

Moreover, pathological analysis was carried out by hematoxylin and eosin (H&E) staining. As shown in Figure 6g, the tissue structure and cell morphology of the necrotic area in the PBS, Amb, and LIQ‐TPA‐TZ + L groups were blurred with a large number of cells dying (blue arrow) on day 1. Large infiltration of neutrophils (black arrow) and lymphocytes (green arrow) was observed. The capillaries were obviously dilated and congested (yellow arrow)Indicating that the oral mucosa was seriously infected. On the 7th day, no significant improvement of these symptoms in the PBS group was recorded. But new capillaries appeared in the infected area of the Amb group. Although most of the blood vessels were dilated, red blood cells exudated, and endothelial cells increased, there was a small amount of lymphocyte infiltration and collagen fiber formation, indicating that the recovery of the Amb group was better than that of the PBS group. In contrast, the infected area of the LIQ‐TPA‐TZ + L group showed abundant new capillaries and collagen fibers formed, indicating that PDT treatment can effectively remove fungi and accelerate mucosal healing. At the same time, the mice in each group were euthanized on the 7th day, and their main organs, heart, liver, spleen, lung and kidney, were obtained. H&E staining was used to detect the biocompatibility of each organ. No obvious physiological morphological changes were observed in the organs of mice in all groups, indicating that LIQ‐TPA‐TZ has good biocompatibility (Figure S10 in Supporting Information S1).

## 3 CONCLUSION

In summary, a mitochondria‐targeting AIE‐active PS, LIQ‐TPA‐TZ, was developed for the photodynamic elimination of *Candida* biofilm and combat oral ulcer in vivo. LIQ‐TPA‐TZ had high ^1^O_2_ and •OH generation efficiency, which is far superior to the most effective and popularly used PS RB. LIQ‐TPA‐TZ can be used for the efficient photodynamic elimination of *C. guilliermondii* after selectively binding with mitochondria through disrupting the respiratory chain, affecting its metabolism and energy supply. Furthermore, it significantly reduced the fungi adhesion and filament growth capabilities, effectively inhibiting the formation of biofilms. Thanks to the ROS sensitizing ability and amphiphilic attribute, it eradicated biofilms, thereby significantly killing *Candida* inside biofilms. We also explored the potential of employing LIQ‐TPA‐TZ‐mediated PDT for treating oral ulcerin in vivo. It can destroy the colonized mycelia, clear the biofilm on the mucosal surface, and accelerate the healing of mucosal defects. LIQ‐TPA‐TZ is expected to have practical applications for candidiasis treatment in the clinic.

## CONFLICT OF INTEREST STATEMENT

The authors declare no conflicts of interest.

## ETHICS STATEMENT

All animal procedures were carried out under the guidelines set by the Institutional Animal Care and Use Committee of Sichuan province, and the overall project protocols were approved by the Animal Ethics Committee of Southwest Jiaotong University.

## Supporting information

Supporting Information S1

## Data Availability

The data that support the findings of this study are available in the supplementary material of this article.
