# Supplementary material for: A mitochondria‐targeted aggregation‐induced emission photosensitizer for eradication Candida bioﬁlms and treating oral ulcer
Source: Smart Mol. 2025 Feb 17;3(3):e20240060. doi: 10.1002/smo.20240060 (PMC12483137; doi:10.1002/smo.20240060)
Supplement: Supplementary file 1 — Supporting Information S1 [file SMO2-3-e20240060-s001.docx]

**Supporting Information**

**A Mitochondria‐Targeted Aggregation-Induced Emission Photosensitizer Eradicate *Candida* Bioﬁlms for Treating Oral Ulcer**

*Kun-Mei Li^1,2^, Yun Wang^2^, Feng-Wei Xia^2^, Shun Feng^2^,* *Xiao-Qi Yu^3^, Ming-Yu Wu**^1,2*^*

K.-M. Liu, Y. Wang, F.-W. Xia, Prof. S. Feng, Prof. X.-Q. Yu and Prof. M.-Y. Wu,

^1^College of Biomedical Engineering, Sichuan University, Chengdu, 610064, China.

^2^School of Life Science and Engineering, Southwest Jiaotong University, Chengdu, 610031, China.

^3^Asymmetric Synthesis and Chiral Technology Key Laboratory of Sichuan Province, Department of Chemistry, Xihua University, Chengdu 610039, P. R. China.

E-mail: [wumy1050hx@swjtu.edu.cn](mailto:wumy1050hx@swjtu.edu.cn).

[1.	Experimental Procedures	3](#_Toc184659907)

[1.1 Materials and General Instruments	3](#_Toc184659908)

[1.2 ROS Detection	3](#_Toc184659909)

[1.3 Hemolytic Activity of Red Blood Cells	4](#_Toc184659910)

[1.4 Cell Counting Kit-8 (CCK-8) assay for cell cytotoxicity	4](#_Toc184659911)

[1.5 Preparation of Pathogen Suspension	4](#_Toc184659912)

[1.6 Photodynamic Anti-Fungi Therapy	5](#_Toc184659913)

[1.7 Biofilm for Mation and Antibiofilm Activity	5](#_Toc184659914)

[1.8 Fungi Staining and Imaging	6](#_Toc184659915)

[1.9 Mitochondrial Colocalization Experiment	6](#_Toc184659916)

[1.10 Scanning Electron Microscopy (SEM) Analysis	6](#_Toc184659917)

[1.11 Flow Cytometric Analysis and Fluorescence Imaging	7](#_Toc184659918)

[1.12 *In Vivo* Anti-Infection Assay	7](#_Toc184659919)

[1.13 Histological Analysis	8](#_Toc184659920)

[1.14 Statistical Analysis	8](#_Toc184659921)

[2.	Supplementary Figures	9](#_Toc184659922)

1. **Experimental Procedures**

## 1.1 Materials and General Instruments

All chemical reagents were obtained from J&K Scientific and were used without further purification. DCF-DA (2’,7’-dichlorofluorescein diacetate) Detection Kit, Sytox Green, 9,10-Anthracenediylbis-(methylene)-dimalonic acid (ABDA), crystal violet, and Rose Bengal (RB) were purchased from Sigma-Aldrich. 2-[6-(4’-Hydroxy)phenoxy-3H-xanthen-3-on-9-yl]benzoic acid (HPF) was purchased from aladdin. Fluorescence spectra were obtained using a Horiba Duetta spectrofluorimeter with a 10 mm quartz cuvette. UV-Vis absorption spectra were recorded on a Hitachi PharmaSpec UV-1900 UV-Visible spectrophotometer. Confocal fluorescence images were recorded using a Nikon A1R+ confocal laser scanning microscope. Zeta-potential and average particle size were recorded using Nano ZS (ZEN3600). The light source was a white LED illuminant from Opple Lighting Co., LTD with the power of 5 W, diameter of 1 cm and intensity of 20 mW cm^-2^.

## 1.2 ROS Detection

A commonly used ROS indicator DCFH-DA was utilized to detect the ROS generation of **LIQ-TPA-TZ** in solution under white light irradiation (20 mW cm^-2^). Briefly, 4 mL DCFH-DA in ethanol (1 mM) was added to 16 mL NaOH solution (10 mM) and stirred at room temperature for 30 minutes. Then the hydrolysate was diluent with 80 mL of PBS and kept in dark at 4 ^o^C. DCF-DA (10 µM) was mixed with PS (10 µM) in PBS under dark and then exposed to light for 0-70 seconds. The fluorescent intensity at 525 nm was monitored by the fluorescence spectrum with the excitation of 488 nm.

The •OH generation measurements were conducted using hydroxyphenyl fluorescein (HPF) as the indicator. PBS buffer solution containing 5 μM HPF (stock solution: 5 mM in DMF) and **LIQ-TPA-TZ** (10 μM) was irradiated with white light irradiation for 0-5 minutes. The fluorescence intensity at 515 nm was recorded with the excitation wavelength at 492 nm.

The ^1^O_2_ generation was measured using ABDA as an indicator, and RB was employed as the standard photosensitizer. ABDA (50 µM) was mixed with the **LIQ-TPA-TZ** or RB (5 µM) in DMSO/water (1:99, *v*/*v*) and exposed to white light illumination for 0-5 minutes (20 mW cm^-2^). The absorbance of ABDA at 378 nm was recorded at different illumination time to obtain the decay rate of the photosensitizing process.

## 1.3 Hemolytic Activity of Red Blood Cells

Prepare a 2% red blood cell (RBC) suspension: Mouse blood was collected in a vacuum blood collection tube coated with sodium heparin, centrifuged at 1200 rcf min^-1^ for 10 minutes. The supernatant was removed, washed three times with PBS, and diluted with PBS to obtain a 2% RBC suspension. 180 μL of the 2% RBC suspension and 20 μL of **LIQ-TPA-TZ** were mixed, and Shaked well to adjust the final concentration to 0.5, 1, 2, 4, 8, and 16 μM. For the blank group and positive control group, the same volume of PBS or Triton X-100 was added, and incubated at 30 ^o^C for 1 hour, centrifuged at 1200 rcf min^-1^ for 5 minutes. The supernatant was collected to measure the OD value at 545 nm. The formula for calculating the percentage of hemolysis is as follows:

**Hemolysis Ratio=** $\frac{\mathbf{OD}\left( \mathrm{Sample} \right)\mathbf{-OD}\left( Negative control \right)}{\mathbf{OD}\left( Positive control \right)\mathbf{-OD}\left( Negative control \right)}\boldsymbol{*}100\%$

## 1.4 Cell Counting Kit-8 (CCK-8) assay for cell cytotoxicity

The standard CCK-8 assay was employed to evaluate the toxicity of the **LIQ-TPA-TZ** to mammalian cells. Approximately 5000 cells per well were seeded in 96-well plates with 100 μL of culture medium and allowed to grow overnight to achieve 70-80% cell confluence. 100 μL fresh medium containing different concentrations of **LIQ-TPA-TZ** (0-16 μM) was then added to the cells and incubated for 30 min. Subsequently, the cells were either shielded from light or exposed to white light (20 mW cm^−2^) for 30 min. After incubated for an additional 24 h, a mixture of 10 μL of CCK-8 solution and 90 μL of the culture medium was added to each well and incubated for additional 1 hour. The absorbance at 450 nm was recorded using an ELISA plate reader. Cell viability was calculated using the formula:

**Cell viability (%)** =$\frac{\mathbf{OD}\left( 450 \mathrm{Sample} \right)\mathbf{-OD}\left( 450 cck8 \right)}{\mathbf{OD}\left( \mathrm{Control} \right)\mathbf{-OD}\left( 450 cck8 \right)}\boldsymbol{*}100\%$

## 1.5 Preparation of Pathogen Suspension

A single *Candida guilliermondii* (*C. guilliermondii*) colony on solid agar plate was added to 10 mL Sabouraud Dextrose Broth (SDB) medium, and then incubated for about 12 hours at 30 ^o^C under the shaking of 200 rpm. After harvested by centrifuging at 8000 rpm min^-1^ for 5 minutes, the *C. guilliermondii* were washed with PBS three times and re-suspended with PBS, and diluted to an optical density (about 10^8^ CFU mL^-1^). The concentrations of the *C. guilliermondii* suspension were determined via optical density measurements of absorbance at 600 nm using a cell density meter.

## 1.6 Photodynamic Anti-Fungi Therapy

*C. guilliermondii* was selected to verify the antifungal effects of **LIQ-TPA-TZ**. 10^8^ CFU mL^-1^ *C. guilliermondii* were incubated with different concentration of **LIQ-TPA-TZ** for 5 minutes at ambient temperature. After washed with PBS, the *C. guilliermondii* were re-dispersed in PBS, and then kept under dark or exposed under white light (20 mW cm^-2^) for 20 minutes. 10 μL *C. guilliermondii* suspension were taken from each sample, serially diluted for 5-fold with PBS, spread on SDA and incubated at 30 ^o^C for 24 hours. The viability was then determined and quantified by the plate count method. Each experiment was performed independently at least three times.

## 1.7 Biofilm for Mation and Antibiofilm Activity

Crystal violet staining method was used to detect the photodynamic ablation of *C. guilliermondii* biofilms. In this experiment, *C. guilliermondii* biofilms were cultured in a six-well plate or a confocal dish.

Culturing *C. guilliermondii* biofilms in the six-well plate: Sterile coverslips were placed in the wells of the six-well plate and filled with SDB liquid medium and *C. guilliermondii suspension*. The plate was then incubated at 30 ^o^C for 72 hours, and the planktonic cells were washed away with PBS.

Culturing *C. guilliermondii* biofilms in the confocal dish: SDB liquid medium and *C. guilliermondii* suspension were added to the confocal dish, incubated at 30 ^o^C for 72 hours, and the planktonic cells were washed away with PBS.

Photodynamic ablation of *C. guilliermondii* biofilms: **LIQ-TPA-TZ** (0, 5, 10, 20, 30 μM) was added to the wells of the six-well plate or the confocal dish, incubated for 30 minutes washed with PBS, re-suspended with PBS. The light-exposed group was placed under white light (20 mW cm^-^²) for 30 minutes, while the other groups were kept in the dark.

Crystal violet assay was conducted to access the eradication of biofilms. SDB medium (150 μL) and *C. guilliermondii* suspension (10 μL) was added to a 96-well plate and incubated at 30 ^o^C for 36 hours. Then, different concentration **LIQ-TPA-TZ** (0, 5, 10, 20, 30 μM) was added and incubated in the dark for 30 minutes. After washed with PBS. The plate was irradiated with light for 30 minutes or kept in the dark. The biofilms were fixed with methanol (100 μL) for 15 minutes, stained with 1% crystal violet solution (100 μL) for 15 minutes. After dissolv the crystal violet with 33% ice-cold acetic acid solution (100 μL), the absorbance at 590 nm was measured using a microplate reader.

MTT assay to access the survival rate of *C. guilliermondii* in biofilms. After different treatment of the biofilm was incubated with MTT (5 mg mL^-1^) 30°C for 3 hours. 150 μL of DMSO was added to each well, and shaked for 15 minutes. Finally, the absorbance at 490 nm was recorded using a microplate reader.

## 1.8 Fungi Staining and Imaging

1000 μL *C. guilliermondii* (OD_600_ = 1.0) suspension in a 1.5 mL centrifuge tube were harvested by centrifuging (8000 rpm min^-1^, 5 minutes). The supernatant was removed and washed with PBS for 3 times. 1000 μL of **LIQ-TPA-TZ** or Amb (4 μM) in PBS solutions were add, incubated for 5 minutes at ambient temperature. After washed with PBS for 3 times, the *C. guilliermondii* were dispersed withed PBS and exposed to white light or kept under dark for 20 minutes. Then, the *C. guilliermondii* were stained with Sytox Green (1 μM) at 30 ^o^C for 10 minutes, washed with PBS for 3 times and dispersed evenly in PBS. For Amb group, the fungus were stained with Hoechst 33342 at 30 ^o^C for 10 minutes, washed with PBS for 3 times and disperse evenly in PBS. The confocal fluorescence images were recorded with Nikon A1R+ laser scanning confocal microscope (Nikon A1R+).

*C. guilliermondii* biofilms grown in a 96-well plate were treated with PBS, **LIQ-TPA-TZ**, **LIQ-TPA-TZ**+L, and Amb (30 μM). After treatment, the biofilms were stained with Sytox Green dye (1 μM) for 10 minutes, centrifuged, washed with PBS, and evenly dispersed in PBS. Laser confocal imaging was then performed.

The 450nm laser and 600–700 nm emission filter was used for **LIQ-TPA-TZ**, 488 nm laser and 500–600 nm emission filter was used for Sytox Green, and 350 nm laser and 400–500 nm emission filter were used for Hoechst 33342, respectively.

## 1.9 Mitochondrial Colocalization Experiment

C*. guilliermondii* were counterstained with LIQ-TPA-TZ (4 μM) and LIQ-3 (4 μM) for 5 minutes. After centrifugation at 8,000 rpm for 5 minutes, the supernatant was discarded and the fungus were washed three times with PBS. Subsequently, the cells were evenly dispersed in PBS for laser confocal imaging.

## 1.10 Scanning Electron Microscopy (SEM) Analysis

The morphologies of *C. guilliermondii* with different treatment were observed by SEM. The fungus was treated with PBS, **LIQ-TPA-TZ**, Amb (4 μM) at 30 ^o^C for 5 minutes, and then kept under dark or exposed under white light for 20 minutes. The *C. guilliermondii* were fixed with 2.5% glutaraldehyde and dehydrated successively by 30%, 50%, 70%, 80%, 90%, 95% and 100% ethanol solution (*v*/*v*) over 10 minutes. Finally, the samples were disposed with metal spraying for SEM analysis (COXEM EM-30).

The biofilms of *C. guilliermondii* treated with PBS, **LIQ-TPA-TZ**, **LIQ-TPA-TZ**+L, and Amb (30 μM) were fixed with 2.5% glutaraldehyde and kept at 4 ^o^C for 12 hours. They were then dehydrated using a gradient of ethanol (30%, 50%, 70%, 85%, 95%, 100%), followed by air-drying and gold sputter coating. Scanning electron microscopy was utilized to observe the morphology of *C. guilliermondii* within the biofilms.

## 1.11 Flow Cytometric Analysis and Fluorescence Imaging

JC-1 staining: 500 µL *C. guilliermondii* (100 µL, OD_600_ = 2.0) suspension was incubated with 0.5 μM of **LIQ-TPA-TZ** in dark, and then irradiated for different time under white light (20 mW cm^-2^). After removed the unbound **LIQ-TPA-TZ** by centrifuging (8000 rpm min^-1^, 5 minutes), the fungu were incubated with 500 µL of 10 μM JC-1 in dark for 15 minutes, centrifuged to remove the supernatant, resuspended with 500 μL of PBS and placed on ice for flow cytometric analysis or Nikon A1R+ laser scanning confocal microscope (Nikon A1R+). The green and red fluorescence signals were collected from FITC and PI channel, respectively.

## 1.12 *In Vivo* Anti-Infection Assay

Healthy female ICR mice (20-25 g) were used. The mice were adapted for 1 week. To decrease the self-limitation of mice toward *C. guilliermondii*, immune-suppressor with prednisolone (125 mg kg^-1^) were intramuscular injected at day 1 before infection. In order to prevent unknown bacterial infection, 0.83 g L^-1^ tetracycline hydrochloride was added to drinking water. Mice were anesthetized with isoflurane. The tongue of mice was burned with 10% NaOH solution for 20 seconds, and then washed with normal saline immediately. To construct the oral mucosal infection model, cotton balls immersed in solutions containing 10^8^ CFU mL^-1^ of *C. guilliermondii* were placed on the tongue of mice for 5 minutes. The control group was orally smeared with PBS.

After the successful establishment of oral ulcer model, the infected mice were randomly divided into 3 groups (PBS group, LIQ-TPA-TZ +L group, Amb group). Uninfected healthy mice were used as a control group (Control group). Mice were anesthetized with isoflurane and their tongues were pulled out of their mouths with a hemostatic forceps. PBS group, LIQ-TPA-TZ +L group and Amb group were infused with 50 μL PBS, LIQ-TPA-TZ (30 μM) and Amb (30 μM), respectively, and incubated in the dark for 30 minutes. The tongue ulcer surface of **LIQ-TPA-TZ**+ L group was irradiated with white light (20 mW cm^-2^) for 30 minutes. Meanwhile, the other 3 groups were placed in the dark for continuous treatment for 7 days. To investigate the therapy effect, photographs, colonies, weights, survival rate, and pathology index of mice were recorded every two day. At day 1, and the Day 7, one mouse in each group was sacrificed, and the tissues of oral mucosa were harvested for pathological analysis with hematoxylin and eosin (H&E) staining.

## 1.13 Histological Analysis

The collected tissues were fixed in 4% paraformaldehyde, and then embedded in paraffin. The fixed tissues were cut into slices with a thickness of 4 mm. Next, hematoxylin-eosin (H&E) staining was carried out according to the standard protocol.

## 1.14 Statistical Analysis

Error bars represent the standard deviation derived from three independent measurements. The statistical significance of differences was determined by a One Way ANOVA, and *P < 0.05, **P < 0.01, and ***P < 0.001 were used to indicate the statistical difference.

# 2. Supplementary Figures

Table S1. Photophysic properties of **LIQ-TPA-TZ** in solvents with different polarity

| **Solvent** | **λ_abs_（nm）** | **ε（M^-1^ cm^-1^）** | **λ_em_（nm）** | **Ф_F_（%）** | **△SS^b^/nm** |
| --- | --- | --- | --- | --- | --- |
| Toluene | 458 | 0.464×10^4^ | 546 | 16.88 | 88 |
| THF | 452 | 0.546×10^4^ | 621 | 15.12 | 169 |
| EtOH | 462 | 0.633×10^4^ | 669 | 4.33 | 207 |
| DMSO | 454 | 0.544×10^4^ | 680 | 2.23 | 226 |
| PBS | 450 | 0.356×10^4^ | 614 | 9.38 | 164 |

Figure S1. PL spectra of DCF-DA (10 μM) in the present of **LIQ-TPA-TZ** (10 μM) under white light irradiation from 0-70 seconds (λ_ex_ = 488 nm). All experiments were repeated three times.

Figure S2. PL spectra of HPF (5 μM) in the present of **LIQ-TPA-TZ** (10 μM) under white light irradiation from 0-5 minutes (λ_ex_ = 492 nm). All experiments were repeated three times.

Figure S3. ^1^O_2_ generation efficiency of **LIQ-TPA-TZ**. UV-vis spectra of ABDA in the presence of **LIQ-TPA-TZ** (A), RB (B). (C) decomposition rates of ABDA in the absence or presence of **PS** under light illumination, where *I*_0_ and *I* are the initial and final absorbances of ABDA at 378 nm, respectively. All experiments were repeated three times.

Figure S4. Biocompatibility of **LIQ-TPA-TZ**. Images (A) and hemolysis ratio of **LIQ-TPA-TZ (B)** (****P < 0.0001). (C) Cell viability of L929 cells after incubated with different concentration of **LIQ-TPA-TZ** and treated with dark or white light (20 mW cm^-2^) irradiation.

Figure S5. SEM images of *C. guilliermondii* after treatment with PBS, **LIQ-TPA-TZ** or Amb (4 μM) in the absence or presence of white light illumination for morphological analysis. White line indicates the scale bar (1 μm).

Figure S6. SEM images of *C. guilliermondii* biofilms after treated with PBS, **LIQ-TPA-TZ** or Amb (30 μM) in the absence or presence of white light illumination for morphological analysis. White line indicates the scale bar (1 μm), black line indicates the scale bar 3 μm).

Figure S7. (A) PCA Principal Component Analysis Diagram. (B) Differential gene statistical map.

Figure S8. Protein-protein interaction network (PPI) diagram.

Table S2. The genes symbol of gene function anotation

| Function | LIQ-TPA-TZ | |
| --- | --- | --- |
|  | up | down |
| Oxidative Stress | *AO-I;*  *ISD11;*  *GUS1;*  *SUI2;*  *CAM1;*  *MRP1;*  *TIF3;*  *MKC1;*  *KAP123;*  *ATX1;*  *TPS1;*  *TYS1;*  *SOD2;*  *HCR1;*  *SLH1;*  *RSM26;*  *TIF4632;*  *PST2;*  *CPA1;*  *NIP1;*  *PRT1;*  *RBG1;*  *ARA1;*  *HGT1;*  *MEP2* |  |
| Drug Reaction |  | *CAP1;*  *SKY1;*  *CRZ1;*  *ROD1;*  *GIN4;*  *TUB1;*  *STP3;*  *ISW2;*  *CLB2;* |
| Filamentous Growth |  | *NIK1;*  *INP53;*  *RBF1;*  *NAG1;*  *HXK1;*  *HST3;*  *FGR15;*  *WOR2;*  *SSO2;*  *SKN7;*  *ASH1;*  *MDS3;*  *SIP2;*  *STE20;*  *STP4;*  *SFL2;*  *KSP1;*  *MSB2;*  *SPA2;*  *SLN1;*  *MTS1;*  *HDA1;*  *ASG1;*  *PDA1;*  *glt1;*  *SKO1;*  *BMT8;*  *HSX11;*  *RFG1;*  *SSU1;*  *MNN2;*  *MEP2;*  *ITR1;* |
| Cell Adhesion |  | *SUN41;*  *AFT2;*  *ALS9;*  *DFI1;*  *BUD4;*  *TRY6;*  *SUC1;*  *SAP9;*  *YWP1;*  *TEC1;*  *ADA2;*  *WAR1;*  *SNF2;*  *BCR1;* |
| Ion Homeostasis |  | *sit2;*  *PHO80;*  *ALA1;*  *MAC1;*  *APE1;*  *SMF1;*  *ATG15;*  *VHC1;*  *TOK1;*  *NHA1;*  *FRE8;*  *KKQ8;*  *PMC1;*  *FET3;*  *MNR2;*  *IZH3;*  *ALR1;*  *MDM31;*  *PER1;*  *YPI1;*  *SMF2;*  *CFL1;*  *FTH1;*  *PPZ1;*  *PHO91;*  *CRF1;*  *PHO2;*  *QRI7;*  *IRA2;*  *REV1;*  *AVT4;*  *VHC1;*  *RNY1;*  *YPT7;* |
| Mitochondrial Correlation | *MTG1;*  *kti12;*  *ARG1;*  *MPC3;*  *nuo-10.5;*  *COR1;*  *PET117;*  *MBA1;*  *ACP1;*  *COQ5;*  *COQ11;*  *FUM1;*  *FUM1;*  *QCR8;*  *DLD2;*  *ARG5;*  *RCF1;*  *nuo20.9;*  *COA6;*  *IDH2;*  *COX8;*  *COX9;*  *COX4;*  *GOR1;*  *PDB1;*  *COX7;*  *MAM33;*  *MIS1;*  *YCP4;*  *HTD2;*  *nuo14.8;*  *GDB1;*  *CBP3;*  *aI2;*  *COB;*  *MSD1;*  *FMP27;*  *LYS21;*  *LEU4;*  *TMA10;*  *BAT2;*  *HNT2;*  *LEU4;*  *AIM18;*  *TUF1;*  *MRX12;*  *ISM1;*  *MTQ1;*  *mrpl9;*  *HER2;*  *MTO1;*  *TRM1;*  *MEF1;*  *VAS1;*  *HTS1;*  *RIM2;*  *COX17;*  *FMP41;*  *MRX8* |  |

Figure S9. Magnified photographs of the oral mucosal infection on the tongue of mice.

Figure S10. Photographs images (A) and H&E staining analysis ((B) of mouse organs (heart, liver, spleen, lungs, kidneys) from the mice with different treatment (Scale bar: 50 μm).
